# Empyema of the Maxillary Antrum

**Published:** 1907-09-21

**Authors:** 


### Laryngology and Rhinology.

### EMPYEMA OF THE MAXILLARY ANTRUM.

The accessory sinuses of the nose present certain peculiar features, and in consequence the problems which confront us in the treatment of suppuration within these cavities are somewhat different from those to be faced in the treatment of inflammation in other parts of the body. Thus the walls of a sinus are bony and cannot contract after evacuation of the pus, as do the walls of an abcess in the soft tissues; and in health it is lined by epithelium, which is more or less destroyed in cases of suppuration. There are only two ways, therefore, by which healing can result; firstly, by regeneration of the epithelium after drainage of the pus, and, secondly, by obliteration of the cavity by surgical removal of a sufficient part of its bony walls. This latter method is possible in the case of the frontal sinus and some of the ethmoidal cells, but it cannot be applied to empyema of the maxillary antrum, the complete obliteration of which would involve the almost entire removal of the upper jaw.

### Some General Principles of Treatment.

It remains, then, to consider the former method. In an acute and quite recent case of suppuration the epithelium will probably not have been extensively destroyed, and simple evacuation of the pus, together with washing out with a mild antiseptic lotion, will sometimes be sufficient to effect a cure; but in cases of longer duration the surfaces denuded of epithelium will heal but slowly, and the discharge will persist for a considerable time, usually for several months, though we can at once prevent it from collecting and decomposing, and the symptoms which result from absorption of the pus will cease after operation, as also the pain due to tension within the cavity. As drainage must be continued for so long a period it is important that the opening be placed where it causes the least possible inconvenience. The cavity of the antrum can be reached by three routes—(1) by the alveolar process through the socket of a tooth, or through the gap where a tooth has been lost, (2) through the canine fossa, or (3) through the nose.

### Drainage.

Drainage through a tooth-socket was for long the routine method of treatment, but it is now much less frequently used by rhinologists, especially since it has been recognised that antral empyema is less often due to dental caries than was formerly supposed, and the method has several grave disadvantages. It is true that the opening is at the lowest part of the cavity, but if an open tube be used the pus trickles continuously into the mouth, and food-particles are carried into the antrum, whilst if a plug be employed the advantages of drainage at the lowest point are lost; also the opening is of necessity small, and does not allow of the evacuation of the solid masses of inspissated pus which are often present. A sound tooth, or one which the dentist can save, should never be sacrificed. In practice the method is unsatisfactory except for recent cases; patients find the tube troublesome, and become weary of it, for the discharge often continues in small amount for an indefinite time; therefore this route should only be used in recent cases of apparently dental origin.

### The Nasal Operation.

Through the canine fossa a large opening can be made, by which the antrum may be thoroughly inspected, but for drainage this route has the same disadvantages as the preceding, in that it opens into the mouth. If this opening be maintained long for packing or syringing it frequently refuses to close, and a permanent fistula remains.

By far the best route for drainage of the antrum is into the nose; as compared with drainage into the mouth, the discharge causes much less inconvenience, and reinfection of the cavity is less likely to occur. Probably the best method, especially for severe and long-standing cases, is by a combination of the canine fossa and nasal routes; in this, the Caldwell-Spicer operation, a large opening is made through the canine fossa, the cavity inspected and cleared of polypi and inspissated pus, and an opening made into the nose. No packing is necessary, and the after-treatment should be carried out through the nose, so that the canine fossa opening may heal at once; this it will readily do, and stitches are unnecessary. The opening into the nose must be very free; in fact, it is better to remove the entire partition between the two cavities, especially the thick ridge along the floor; if this be done simple syringing of the nose will suffice to thoroughly clean the antrum, whereas if the opening be smaller, the patient must be taught to pass a curved cannula into the cavity. The lining of the antrum should not be scraped away, for it is only by regeneration of the epithelium that a complete cure results.

### The Newest Method.

The nasal route has frequently been used alone; in acute recent cases, such as occur in the course of influenza, the antrum may be tapped through the inferior meatus with a fine trocar and washed out; this can easily be done under cocaine, and may result in cure at once or after repetition, but it often fails, and it should be remembered that an apparently acute case is not infrequently merely an exacerbation of chronic disease. Recently a method has been introduced, which consists in removing a large part of the inner wall of the antrum through the nose with angular knives or cutting-forceps. The operation requires general anæsthesia, but the soreness about the lips and cheek which results from opening the canine fossa is prevented; the cavity can be explored with the little finger and curetted if necessary, but it cannot be so thoroughly examined. The method has given very good results, but the opening in the canine fossa will probably still be preferred for the more severe and chronic cases.

